# Cardiovascular disease in a nationwide population of Danish women with polycystic ovary syndrome

**DOI:** 10.1186/s12933-018-0680-5

**Published:** 2018-03-08

**Authors:** Dorte Glintborg, Katrine Hass Rubin, Mads Nybo, Bo Abrahamsen, Marianne Andersen

**Affiliations:** 1 Department of Endocrinology, Odense University Hospital, Kløvervænget 6, 3rd Floor, 5000 Odense C, Denmark; 2 Institute of Clinical Research, University of Southern Denmark, 5000 Odense, Denmark; 3 OPEN-Odense Patient Data Explorative Network, Institute of Clinical Research, University of Southern Denmark, 5000 Odense, Denmark; 4 Department of Clinical Biochemistry and Pharmacology, Odense University Hospital, 4800 Odense, Denmark; 5 Department of Medicine, Holbæk Hospital, Holbæk, Denmark

**Keywords:** Polycystic ovary syndrome, Register-based, ICD-10, HT, Cardiovascular disease, Medicine prescriptions, Nationwide

## Abstract

**Background:**

Polycystic ovary syndrome (PCOS) is associated with obesity and low grade inflammation and the risk for cardiovascular disease (CVD) could be increased in PCOS.

**Methods:**

National register-based study including women with PCOS and no previous diagnosis of CVD, hypertension, or dyslipidemia. PCOS Denmark (N = 18,112) included women with PCOS in the Danish National Patient Register. PCOS Odense University Hospital (OUH, N = 1165) was an embedded cohort including premenopausal women with PCOS and clinical and biochemical examination. Three age-matched controls were included per patient in PCOS Denmark (N = 52,769). The main study outcome was CVD events including hypertension and dyslipidemia defined according to nationwide in- and outpatient hospital contact diagnosis codes and/or inferred from filled medicine prescriptions.

**Results:**

The age at inclusion was median (quartiles) 29 (23–35) years and follow up was 11.1 (6.9–16.0) years. The Hazard ratio (95% CI) for development of CVD in PCOS Denmark was 1.7 (1.7; 1.8) (P < 0.001) and the total event rate of CVD was 22.6 per 1000 patient years in PCOS Denmark vs. 13.2 per 1000 patient years in controls (P < 0.001). The median age at diagnosis of CVD was 35 (28–42) years in PCOS Denmark vs. 36 (30–43) years in controls (P < 0.001). Obesity, diabetes, and infertility, and previous use of oral contraceptives were associated with increased risk of development of CVD in PCOS Denmark (P < 0.001). Women in PCOS OUH resembled women in PCOS Denmark regarding risk of CVD. Age, BMI, blood pressure, lipid status, and glycemic status predicted development of CVD in PCOS OUH.

**Conclusion:**

The event rate of CVD including hypertension and dyslipidemia was higher in PCOS compared to controls. The risk of developing CVD must be considered even in young women with PCOS.

**Electronic supplementary material:**

The online version of this article (10.1186/s12933-018-0680-5) contains supplementary material, which is available to authorized users.

## Introduction

The definition of polycystic ovary syndrome (PCOS) includes irregular ovulation, hyperandrogenism, and/or polycystic ovaries when other etiologies are excluded [1]. The pathogenesis of PCOS includes insulin resistance and low grade inflammation [2], and women with PCOS have increased risk of metabolic syndrome [3, 4]. Insulin resistance and low grade inflammation in PCOS could be associated with increased risk of development of cardiovascular disease (CVD). The odds ratio (OR) for CVD in PCOS varied from 1.3 to 2.0 in recent meta-analyses [5–8], but two of the studies reported no statistically significant association between PCOS and CVD [5, 6]. The absolute risk of CVD in premenopausal women is low and previous studies may have had too little power to detect increased risk of CVD in PCOS [6]. Furthermore, several risk factors modify the metabolic risk in PCOS. Obesity is closely related to insulin resistance and inflammation [9]. Lean women with PCOS did not have an increased risk of developing type 2 diabetes compared to age-matched controls [4, 10], and the risk for developing CVD in lean women with PCOS needs to be determined. It is recommended that all women with PCOS should be screened for metabolic syndrome upon diagnosis [11], but measurement of lipids in young women with PCOS rarely changed clinical care [12]. Also the importance of individual risk markers such as high blood pressure (BP), dyslipidemia, and elevated blood glucose for future development of CVD in PCOS remains to be established. The risk of CVD could also be modified by medical treatment of PCOS. Fasting insulin levels were unchanged during treatment with oral contraceptives (OCP) in PCOS [13], but treatment with OCP has been reported to be associated with weight gain [14], increased thrombin generation [15] and increased risk of venous thromboembolism [16, 17], and use of OCP could therefore have an adverse effect on CVD risk.

The aim of the present register-based study was to investigate the risk of developing CVD in women with PCOS and possible modifying effects of obesity, comorbidity, and prescription of OCP. Possible associations between baseline metabolic risk profile and later development of CVD were investigated in a well-described representative subgroup of patients with hyperandrogenism and/or PCOS.

## Materials and methods

**Fig. 1:**
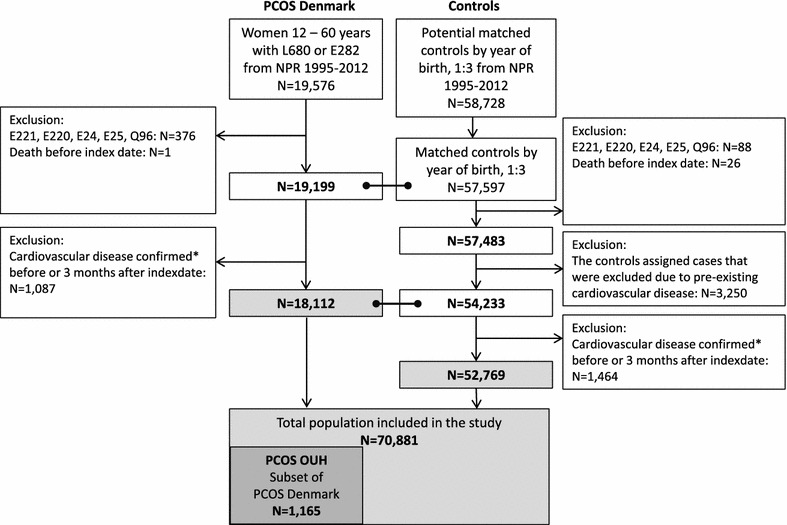
Flowchart of included women with PCOS and controls. CVD was defined as at least one of the following criteria: (1) the presence of a CVD diagnosis in NPR according to ICD10: E78 (dyslipidemia), G45–G46 (TCI, stroke), I10–13 (essential hypertension and hypertension with complications), I20 (angina), I21–25 (myocardial infarction and ichemic coronary disease), I26 (lung embolism), I50 (incompensatio cordis), I63–I64 (cerebral infarction), I65–I66 (occlusion of vertebral and cerebral arteries), I80–82 (venous thrombosis) or (2) prescription of drugs for treatment of CVD according to the National Prescriptions Registry database: antithrombotics (B01), antihypertensives (C02: alpha-blockers, C07: beta-blockers, C08: calcium-antagonists, C09: renin-angiotensin blockers), C10: antilipids. Two or more medicine withdrawals should occur within the same ATC group to be included

The study design and baseline data for this study have recently been reported in detail [18] and data regarding development of type 2 diabetes and incident fractures [10, 19] in the study cohort has recently been published. We used an observational register-based cohort drawn from Danish national health registers including two patient populations with PCOS and one control population (Fig. 1). PCOS Denmark included all women in Denmark aged 12–60 years, who were diagnosed through a hospital contact with PCOS (E282) and/or hirsutism (L680) between January 1st 1995 and the end of 2012. PCOS Odense University Hospital (OUH) included an embedded local sub-cohort of women with PCOS and/or hirsutism treated at OUH with available clinical and biochemical information. For each patient in PCOS Denmark (and in PCOS OUH), three control women born in the same year as the patient were randomly drawn from the civil population register. Controls were assigned the index date (date of first PCOS diagnosis) of their matched PCOS cases and should be alive at the index date of their PCOS case.

### Assays in PCOS OUH

These have been described recently [10, 18] and are presented in the Additional file 1. During 1997–2003, an oral glucose tolerance test (OGTT) was part of the routine evaluation program for newly referred women with PCOS [20].

### The Danish health registries

All Danish individuals are assigned a unique personal identification number and data from national registers can therefore be linked at an individual level. We retrieved information about hospital contacts and filled medicine prescriptions along with dates of death if applicable, for women with PCOS and control subjects from National Patient Register (NPR), the National Prescription Registry and the National Cause of Death Register from 1995 to December 31st 2015.

### Outcome

The primary outcome was CVD defined as at least one of the following criteria: (1) the presence of a CVD diagnosis in NPR according to ICD10: E78 (dyslipidemia), G45–G46 (TCI), I10–13 (essential hypertension and hypertension with complications), I20 (angina), I21–25 (myocardial infarction and ichemic coronary disease), I26 (lung embolism), I50 (incompensatio cordis), I63–I64 (cerebral infarction), I65–I66 (occlusion of vertebral and cerebral arteries), I80–82 (venous thrombosis) or (2) prescription of drugs for treatment of CVD according to the National Prescriptions Registry database: antithrombotics (B01), antihypertensives (C02: alpha-blockers, C07: beta-blockers, C08: calcium-antagonists, C09: renin-angiotensin blockers), C10: antilipids. Patients should be medically treated for a certain time period and therefore, two or more medicine withdrawals should occur within the same ATC group to be included.

#### Secondary outcome

The risk of CVD excluding hypertension and dyslipidemia. This outcome was defined as at least one of the following criteria: (1) the presence of a CVD diagnosis in NPR according to ICD10: G45–G46 (TCI), I20 (angina), I21–25 (myocardial infarction and ichemic coronary disease), I26 (lung embolism), I50 (incompensatio cordis), I63–I64 (cerebral infarction), I65–I66 (occlusion of vertebral and cerebral arteries), I80–82 (venous thrombosis), or (2) prescription of drugs for treatment of CVD according to the National Prescriptions Registry database: antithrombotics (B01). Patients should be medically treated for a certain time period and therefore, two or more medicine withdrawals should occur within the same ATC group to be included.

### Exclusion criteria

Participants with CVD events occurring before and up till 3 months after the index date were excluded. Furthermore, women with the diagnoses E221 (hyperprolactinemia), E220 (acromegaly), E24 (Cushing’s syndrome), E25 (adrenogenital syndrome), and Q96 (Turner syndrome) were excluded from all study cohorts.

### Exposure

A baseline diagnosis of obesity (E66), type 1 and type 2 diabetes and gestational diabetes (E10, E13, E11, E14, O24), infertility (N97, Z350), presence of comorbidity (defined as Charlson index ≥ 1), and use of OCP were used as exposures. Use of OCP [ATC codes G03AA and G03AB (combined progesterone and estrogen OCP), and G03HB01 (OCP containing cyproterone)] was defined as ≥ 2 dispenses of prescriptions in the National Prescription Registry (a minimum of 6 months treatment with OCP). The Charlson Comorbidity Index is based on 19 comorbid conditions [21] and was calculated from the ICD-10 operationalization by Quan et al. [22]. All covariates were defined before the index date.

### Statistical analyses

Descriptive analyses for categorical variables were presented as frequencies and difference between PCOS and controls was evaluated by Chi square test. Continuous variables were tabulated as medians (with quartiles, Q1 and Q3) and nonparametric test on the equality of medians were used to test for differences between groups. P values below 0.05 were considered statistically significant.

Cox proportional hazard models were used to calculate incidence rates, hazard ratios (HR) and 95% confidence intervals (95% CIs) and corresponding P values for outcomes. The regression analyses including PCOS Denmark and controls with CVD as the outcome were carried out crude and adjusted for one of the exposures obesity, diabetes, infertility, comorbidity and use of OCP, and finally adjusted for all four variables: Obesity, diabetes, infertility and use of OCP. The diagnosis codes for diabetes and CVD are included as part of the Charlson index and therefore we did not adjust for Charlson score in the regression models. Regression analyses were repeated with CVD excluding hypertension and dyslipidemia as outcomes. The matching of PCOS and controls was taken into account by estimating stratified baseline hazard for each matching set.

The Cox proportional hazard analyses (regression analyses) in the PCOS OUH cohort were carried out crude and adjusted for age and BMI. Analyses were conducted using STATA 14 (StataCorp 2015). Data were anonymized according to Danish law and regulations, and therefore analyses were performed through a remote VPN access to Statistics Denmark.

### Ethics

The core study was an open register-based cohort study. The study did not need approval from the local Ethics committee or Institutional Review Board by Danish law. The study was approved by the Data Protection Agency and by Statistics Denmark, Project No. 704175.

## Results

The flow chart of included women is shown in Fig. 1. A total of 18,112 women with PCOS (PCOS Denmark and the embedded cohort PCOS OUH, N = 1165) and 52,769 controls were included in the study.

### PCOS vs. controls (Additional file 1: Appendix Table S1)

The median age of women in PCOS Denmark was 29 years. By definition, the age was comparable in PCOS Denmark vs. controls. Women in PCOS Denmark had higher prevalence of ICD10 codes and medicine prescriptions related to the metabolic syndrome occurring before the index date compared to controls. The prevalence of comorbidity and infertility was higher in PCOS and the prescription of OCP and drugs for fertility treatment and the number of births before the index date (23% vs. 19% had ≥ 1 births) was higher in PCOS than controls.

Women in PCOS Denmark resembled women in PCOS OUH regarding age and ICD10 codes for the metabolic syndrome and comorbidity before the index date, but they had higher average number of births, lower prevalence of infertility diagnoses and lower prevalence of antidiabetic prescriptions.

### Baseline clinical and biochemical data in PCOS OUH (Additional file 1: Appendix Table S2)

The median age of women in PCOS OUH was 29 years, BMI was 27.0 kg/m^2^, 52% had waist ≥ 88 cm, 61% had BMI ≥ 25 kg/m^2^, 38% had elevated systolic BP, 34% had elevated diastolic BP, and 18% had TG ≥ 1.7 mmol/L.

### CVD event rates (Table 1)

The median (Q1–Q3) follow up duration was 11.1 (6.9–16.0) years. The incidence rate of CVD was 22.6/1000 person years (PY) in PCOS OUH, 22.0/1000 PY in PCOS Denmark, and 13.2/1000 PY in controls (P < 0.001 PCOS Denmark vs. controls). The incidence rate for CVD excluding hypertension and dyslipidemia was 6.4/1000 PY in PCOS Denmark vs. 4.5/1000 PY in controls (P < 0.001).

**Table 1:** Event rates of CVD in PCOS OUH, PCOS Denmark and controls

	PCOS OUH (N = 1159)		PCOS Denmark (N = 17,995)		Controls (N = 52,329)		P^a^	P^b^
	N (%)	IR	N (%)	IR	N (%)	IR		
CVD events	264 (23)	22.6	3970 (22)	22.0	7344 (14)	13.2	< 0.001	0.54
CVD (HT and DL excluded)	71 (6)	5.4	1290 (7)	6.4	2678 (5)	4.5	< 0.001	0.16
ICD10 CVD, total	121 (10)	9.5	1727 (10)	8.7	3089 (6)	5.2	< 0.001	0.31
E 78 (dyslipidemia)	33 (3)	2.5	431 (2)	2.1	653 (1)	1.1	< 0.001	0.30
G45–46, I63–66 (TCI, stroke)	16 (1)	1.2	206 (1)	1.0	524 (1)	0.9	0.102	0.44
I10–13 (hypertension)	77 (7)	5.9	1004 (6)	4.9	1484 (3)	2.5	< 0.001	0.10
I20–25 (CVD)	20 (2)	1.5	364 (2)	1.8	854 (2)	1.4	0.001	0.46
I26 (VTE lung)	4 (0.4)	0.3	97 (0.5)	0.5	151 (0.3)	0.2	< 0.001	0.35
I50 (incompensatio)	0	Na	47 (0.3)	0.2	200 (0.4)	0.3	0.018	0.07
I80–82 (VTE extremities)	17 (1)	1.3	216 (1)	1.0	451 (1)	0.7	< 0.001	0.39
Medical treatment	247 (21)	20.8	3643 (20)	20.0	6656 (13)	11.9	< 0.001	0.35
Antithrombotics (B01)	50 (4)	3.8	940 (5)	4.6	1887 (4)	3.2	< 0.001	0.15
Antihypertensive (C02, C07–09)	189 (16)	15.4	2744 (15)	14.5	5087 (10)	8.9	< 0.001	0.30
Antilipids (C10)	121 (10)	9.4	1601 (9)	8.1	2833 (5)	4.8	< 0.001	0.06

IR: incidence rate. Presented per 1000 PY (person years)

VTE: venous thromboembolism

HT: hypertension defined as ICD10 codes I10–13 OR prescription of C02, C07–09 (two or more medicine withdrawals within the same ATC group 3 months or later after the index date)

DL: dyslipidemia defined as (ICD 10 code) E78 OR prescription of C10 (two or more medicine withdrawals within the same ATC group 3 months or later after the index date)

P^a^: Chi square test between PCOS Denmark and controls

P^b^: Chi square test between PCOS OUH and controls

### Baseline characteristics according to subsequent development of CVD in PCOS OUH (Table 2)

Women in PCOS OUH who developed CVD were significantly older, more obese (higher BMI and waist circumference), had higher BP (systolic and diastolic), a more adverse lipid profile (LDL, cholesterol, and triglycerides), higher glucose and insulin levels (HbA1c, fasting insulin and glucose, and HOMA-ir), and lower prolactin levels at baseline compared to women in PCOS OUH and no development of CVD, whereas testosterone levels (free and total) and SHBG were comparable in the two groups. Development of CVD was dependent of baseline metabolic risk profile (BMI, waist, systolic and diastolic BP, and triglycerides), but development of CVD was also observed in women with PCOS and BMI < 25 kg/m^2^, waist < 88 cm, systolic BP < 130 mmHg, diastolic BP < 85 mmHg and triglycerides < 1.7 mmol/L.

**Table 2:** Baseline clinical and biochemical characteristics according to subsequent development of CVD in PCOS OUH

	Development of CVD				P^#^
	Yes N = 264		No N = 895		
Baseline characteristics	N (%)	Median (Q1–Q3)	N (%)	Median (Q1–Q3)	
Age (years)	230 (100)	35 (28–39)	935 (100)	27 (21–33)	< 0.001
BMI (kg/m^2^)	245 (93)	30.2 (25.1–35,2)	831 (93)	26.1 (22.6–31.1)	< 0.001
Waist (cm)	134 (51)	100 (84–110)	587 (66)	87 (77–100)	< 0.001
Systolic BP (mmHg)	148 (56)	130 (120–140)	624 (70)	123 (114–131)	0.002
Diastolic BP (mmHg)	148 (56)	85 (78–92)	650 (70)	80 (70–86)	< 0.001
LDL cholesterol (mmol/L)	184 (70)	3.2 (2.6–3.9)	663 (74)	2.6 (2.1–3.1)	0.275
HDL cholesterol (mmol/L)	186 (70)	1.3 (1.1–1.6)	663 (74)	1.4 (1.1–1.6)	< 0.001
Cholesterol (mmol/L)	186 (70)	5.2 (4.5–5.9)	674 (75)	4.5 (4.0–5.1)	< 0.001
Triglycerides (mmol/L)	186 (70)	1.3 (0.9–1.9)	662 (74)	1.0 (0.7–1.3)	0.026
Prolactin (µg/L)	223 (85)	6 (5–9)	731 (82)	7 (5–10)	0.010
HbA1c (mmol/mol)	108 (41)	37 (33–42)	463 (52)	33 (31–37)	0.298
Fasting blood glucose (mmol/L)	150 (57)	4.7 (4.2–5.0)	384 (43)	4.6 (4.3–5.0)	0.012
2 h blood glucose (mmol/L)	149 (56)	6.4 (5.3–7.6)	373 (42)	6.0 (5.2–6.9)	0.001
Fasting insulin (pmol/L)	157 (60)	60 (40–114)	410 (46)	53 (36–82)	0.002
HOMA-ir (pmol mmol/L^2^)	152 (58)	14.8 (9.1–25.9)	388 (43)	11.3 (7.6–17.1)	0.330
Total testosterone (nmol/L)	170 (64)	1.7 (1.2–2.3)	655 (73)	1.8 (1.3–2.5)	0.708
SHBG (nmol/L)	244 (92)	45 (30–64)	829 (93)	44 (31–67)	0.367
Free testosterone (nmol/L)	167 (63)	0.032 (0.021–0.049)	644 (72)	0.033 (0.021–0.050)	0.012
					P^¤^
BMI ≥ 25 kg/m^2^	186 (76)		469 (56)		< 0.001
BMI < 25 kg/m^2^	59 (24)		362 (44)		
Waist ≥ 88 cm	94 (71)		277 (47)		< 0.001
Waist < 88 cm	40 (30)		310 (53)		
Systolic BP ≥ 130 mmHg	84 (57)		205 (33)		< 0.001
Systolic BP < 130 mmHg	64 (43)		420 (67)		
Diastolic BP ≥ 85 mmHg	77 (52)		186 (30)		< 0.001
Diastolic BP < 85 mmHg	71 (48)		438 (70)		
Triglycerides ≥ 1.7 mmol/L	63 (34)		93 (15)		< 0.001
Triglycerides < 1.7 mmol/L	123 (66)		569 (86)		

Data presented as median (Q1–Q3)

*BP* blood pressure, *LDL* low density lipoprotein, *HDL* high density lipoprotein, *2* *h* 2 hours (during oral glucose tolerance test), *HOMA-ir* homeostasis model assessment of insulin resistance, *SHBG* Sex hormone-binding globulin

P^#^: non-parametric test on the equality of medians

P^¤^: Chi squared test

### Baseline characteristics according to development of CVD in PCOS Denmark and controls (Table 3): women with development of CVD including hypertension and dyslipidemia in PCOS Denmark vs. controls

The median age at diagnosis of CVD was significantly lower in PCOS Denmark vs. controls (35 vs. 36 years, P < 0.001) and 90% vs. 88% were aged < 50 years at CVD diagnosis, respectively (P < 0.001). The diagnoses obesity, diabetes, infertility, presence of comorbidity, and prescription of OCP were significantly more prevalent in women with PCOS and development of CVD compared to controls with development of CVD. When hypertension and dyslipidemia was excluded from the diagnosis of CVD, the median age of diagnosis was 36 vs. 38 years in PCOS vs. controls (P < 0.001).

**Table 3:** Characteristics according to development of CVD in PCOS Denmark and controls

	Development of CVD in PCOS			Development of CVD in controls			PCOS vs. controls	P A vs. B	P A vs. C
	Total	Yes A	No B	Total	Yes C	No D			
Development of CVD	17,995	3970	14,025	52,329	7344	44,985			
Age at diagnosis (years) Median (Q1, Q3)	29 (23–35)	35 (28–42)	28 (22–33)	29 (23–35)	36 (30–43)	28 (23–33)	0.009	< 0.001	< 0.001
	N (%)	N (%)	N (%)	N (%)	N (%)	N (%)			
Age < 50 years	17,331 (96)	3591 (90)	13,740 (98)	50,718 (97)	6496 (88)	44,222 (98)	< 0.001	< 0.001	0.001
Baseline characteristics									
Obesity	2005 (11)	444 (11)	1561 (11)	648 (1)	121 (2)	527 (1)	< 0.001	0.924	< 0.001
Diabetes	448 (3)	198 (5)	250 (2)	367 (1)	163 (2)	204 (1)	< 0.001	< 0.001	< 0.001
Infertility	4158 (23)	751 (19)	3407 (24)	2007 (4)	259 (4)	1748 (4)	< 0.001	< 0.001	< 0.001
Comorbidity	796 (4)	215 (5)	581 (4)	1646 (3)	253 (3)	1393 (3)	< 0.001	0.001	< 0.001
Oral contraceptives	10,432 (58)	1752 (44)	8680 (62)	16,005 (31)	1652 (23)	14,353 (32)	< 0.001	< 0.001	< 0.001
Development of CVD (HT and DL excluded)	17,995	1290	16,705	52,329	2678	49,651			
Age at diagnosis (years) Median (Q1, Q3)	29 (23–35)	36 (29–44)	29 (23–35)	29 (23–35)	38 (31–46)	28 (23–34)	0.009	< 0.001	< 0.001
	N (%)	N (%)	N (%)	N (%)	N (%)	N (%)			
Age < 50 years	17,331 (96)	1098 (85)	16,233 (97)	50,718 (97)	2235 (83)	48,4834 (98)	< 0.001	< 0.001	0.182
Baseline characteristics									
Obesity	2005 (11)	128 (10)	1877 (11)	648 (1)	39 (1)	609 (1)	< 0.001	0.149	< 0.001
Diabetes	448 (3)	60 (5)	388 (2)	367 (1)	57 (2)	310 (1)	< 0.001	< 0.001	< 0.001
Infertility	4158 (23)	217 (17)	3941 (24)	2007 (4)	95 (4)	1912 (4)	< 0.001	< 0.001	< 0.001
Comorbidity	796 (4)	98 (8)	698 (4)	1646 (3)	105 (4)	1541 (3)	< 0.001	< 0.001	< 0.001
Oral contraceptives	10,432 (58)	497 (39)	9935 (60)	16,005 (31)	513 (19)	15,492 (31)	< 0.001	< 0.001	< 0.001

Comorbidity was defined as a Charlson index ≥ 1

ICD 10 codes OCP (oral contraceptives): G03AA, G03AB, G03HB01

Hypertension (HT) defined as ICD10 codes I10–13 OR prescription of C02, C07–09

Dyslipidemia (DL) defined as (ICD 10 code) E78 OR prescription of C10

Two or more medicine withdrawals should occur within the same ATC group 3 months or later after the index date)

Diabetes defined as type 1 or type 2 diabetes or gestational diabetes according to ICD10: E10, E11, E13, E14, O24

P values obtained with Chi squared test for categorical variables and non-parametric test on the equality of medians for continuous variables

### PCOS Denmark and development of CVD yes vs. no

Women in PCOS Denmark who developed CVD were significantly older than women in PCOS Denmark without development of CVD (35 vs. 28 years, P < 0.001), the diagnoses obesity, diabetes, and presence of comorbidity were more common, whereas a diagnosis of infertility and use of OCP was lower. When hypertension and dyslipidemia was excluded from the diagnosis of CVD, the diagnosis obesity had similar prevalence at baseline irrespective of later CVD diagnosis (P = 0.15).

### Proportional hazard regression analyses

The HR for development of CVD was 1.7 (1.6; 1.8) in PCOS Denmark vs. controls (Table 4). The HR for CVD excluding hypertension and dyslipidemia was 1.4 (1.3; 1.5). In regression models, a diagnosis of obesity, diabetes, infertility, and a Charlson index ≥ 1 at baseline was associated with a higher risk of CVD. Similar findings were found for CVD excluding hypertension and dyslipidemia. Prescription of OCP at baseline was associated with increased risk of CVD, but did not predict CVD excluding hypertension and dyslipidemia.

**Table 4:** Crude and adjusted Hazard ratios in PCOS Denmark (N = 18,112) and controls (N = 52,769) and development of CVD

	Crude HR (95% CI)	Adjusted HR HR (95% CI)	Adjusted HR HR (95% CI)	Adjusted HR HR (95% CI)	Adjusted HR HR (95% CI)	Adjusted HR HR (95% CI)	Adjusted HR HR (95% CI)
Outcome: CVD							
PCOS (yes/no)	1.7 (1.7; 1.8) P < 0.001	1.6 (1.5; 1.7) P < 0.001	1.7 (1.6; 1.8) P < 0.001	1.7 (1.6; 1.8) P < 0.001	1.7 (1.6; 1.7) P < 0.001	1.7 (1.6; 1.8) P < 0.001	1.6 (1.5; 1.6) P < 0.001
Predictors							
Obesity		2.7 (2.4; 3.1) P < 0.001					2.4 (2.1; 2.7) P < 0.001
Diabetes (yes/no)			4.9 (4.1; 5.9) P < 0.001				4.4 (3.6; 5.3) P < 0.001
Infertility (yes/no)				1.2 (1.1; 1.3) P = 0.001			1.0 (0.9; 1.1) P = 0.802
Comorbidity					1.6 (1.4; 1.8) P < 0.001		
OCP (yes/no)						1.1 (1.1; 1.2) P < 0.001	1.1 (1.1; 1.2) P < 0.001
Outcome: CVD (HT and DL excluded)							
PCOS (yes/no)	1.4 (1.3; 1.5) P < 0.001	1.3 (1.2; 1.4) P < 0.001	1.4 (1.3; 1.5) P < 0.001	1.4 (1.3; 1.5) P < 0.001	1.4 (1.3; 1.8) P < 0.001	1.4 (1.3; 1.5) P < 0.001	1.3 (1.2; 1.4) P < 0.001
Predictors							
Obesity		2.4 (1.9; 3.1) P < 0.001					2.1 (1.7; 2.7) P < 0.001
Diabetes (yes/no)			2.9 (2.2; 3.8) P < 0.001				2.5 (1.9; 3.3) P < 0.001
Infertility (yes/no)				1.3 (1.1; 1.5) P = 0.001			1.2 (1.0; 1.4) P = 0.019
Comorbidity					1.8 (1.5; 2.1) P < 0.001		
OCP (yes/no)						1.0 (0.9; 1.1) P = 0.661	1.0 (0.9; 1.1) P = 0.687

Predictors for development of CVD in PCOS Denmark and controls

Comorbidity was defined as a Charlson index ≥ 1

Hazard ratios are presented for crude models and models corrected for obesity, diabetes, infertility, and use of OCP at the index date

Diabetes defined as type 1 or type 2 diabetes or gestational diabetes according to ICD10s (E10, E11, E13, E14, O24)

In PCOS OUH, age, obesity (BMI, waist), BP (systolic and diastolic), lipid status (LDL, cholesterol, TG), prolactin, blood glucose (fasting, 2 h), insulin (fasting, HOMA-ir) were significant predictors for development of CVD (Table 5). When models were corrected for age and BMI; diastolic BP, lipid status (LDL, cholesterol, TG), and 2 h BG were the best predictors of development of CVD. Similar significant results were found for CVD excluding hypertension and dyslipidemia.

**Table 5:** Crude and adjusted hazard ratios in PCOS OUH and development of CVD

	CVD			CVD (HT and DL excluded)		
	Crude HR (95% CI)	n	Age and BMI Adjusted HR^a^ (95% CI)	Crude HR (95% CI)	n	Age and BMI Adjusted HR^a^ (95% CI)
Age (years)	1.1 (1.1; 1.1) P < 0.001	1076	1.1 (1.1; 1.1) P < 0.001	1.1 (1.1; 1.1) P < 0.001	1076	1.1 (1.1; 1.1) P < 0.001
BMI (kg/m^2^)	1.1 (1.0; 1.1) P < 0.001	1076	1.1 (1.1; 1.1) P < 0.001	1.1 (1.0; 1.1) P < 0.001	1076	1.1 (1.1; 1.1) P < 0.001
Waist (cm)	1.0 (1.0;1.0) P < 0.001	710	1.0 (1.0; 1.0) P = 0.02	1.0 (1.0;1.0) P = 0.004	710	1.0 (1.0; 1.1) P = 0.61
Systolic BP (mmHg)	1.0 (1.0; 1.1) p < 0.001	730	1.0 (1.0; 1.0) p < 0.001	1.0 (1.0; 1.1) P = 0.001	730	1.0 (1.0; 1.0) P = 0.09
Diastolic BP (mmHg)	1.1 (1.0; 1.1) P < 0.001	729	1.0 (1.0; 1.1) P < 0.001	1.1 (1.0; 1.1) P < 0.001	729	1.0 (1.0; 1.1) P = 0.06
LDL (mmol/l)	1.8 (1.6; 2.1) P < 0.001	804	1.5 (1.2; 1.7) P < 0.001	1.3 (1.0; 1.8) P = 0.06	804	1.1 (0.7; 1.5) P = 0.78
Cholesterol (mmol/L)	1.8 (1.6; 2.1) P < 0.001	815	1.5 (1.3; 1.7) P < 0.001	1.4 (1.0; 1.8) P < 0.02	815	1.1 (0.8; 1.5) P = 0.41
Triglycerides (mmol/L)	1.7 (1.4; 2.0) P < 0.001	805	1.5 (1.2; 1.7) P < 0.001	1.3 (0.9; 1.8) P = 15	805	1.1 (0.7; 1.6) P = 0.63
Prolactin (µg/L)	0.96 (0.9; 1.0) P = 0.02	891	0.99 (0.95; 1.02) P = 0.61	1.0 (0.9; 1.1) P = 0.89	891	1.0 (1.0; 1.1) P = 0.33
HbA1c (mmol/mol)	1.0 (1.0; 1.0) P = 0.30	525	1.0 (1.0; 1.0) P = 0.73	1.0 (1.0; 1.0) P = 0.64	525	1.0 (1.0; 1.0) P = 0.81
Fasting blood glucose (mmol/L)	1.5 (1.3; 1.8) P < 0.001	510	1.3 (1.1; 1.5) P = 0.001	1.5 (1.2; 2.0) P = 0.001	510	1.2 (1.0; 1.7) P = 0.15
2 h blood glucose (mmol/L)	1.2 (1.1; 1.3) P < 0.001	497	1.1 (1.1; 1.2) P < 0.001	1.2 (1.1; 1.3) P = 0.003	497	1.1 (1.0; 1.2) P = 0.12
Fasting insulin (pmol/L)	1.0 (1.0; 1.0) P < 0.001	533	1.0 (1.0; 1.0) P = 0.007	1.0 (1.0; 1.0) P = 0.32	533	1.0 (1.0; 1.0) P = 0.82
HOMA-ir (µg/L)	1.0 (1.0; 1.0) P < 0.001	509	1.0 (1.0; 1.0) P = 0.001	1.0 (1.0; 1.0) P = 0.13	509	1.0 (1.0; 1.0) P = 0.81
Total testosterone (nmol/L)	0.9 (0.8; 1.1) P = 0.25	772	1.0 (0.9; 1.2) P = 0.99	0.9 (0.7; 1.3) P = 0.72	772	1.0 (0.8; 1.4) P = 0.89
SHBG (nmol/L)	1.0 (1.0; 1.0) P = 0.43	1000	1.0 (1.0; 1.0) P = 0.30	1.0 (1.0; 1.0) P = 0.77	1000	1.0 (1.0; 1.0) P = 0.23
Free testosterone (nmol/L)	0.75 (0.01; 39.37) P = 0.89	759	1.9 (0.03; 112.8) P = 0.75	0.0 (0.0; 338.2) P = 0.26	759	0.0 (0.0; 1858.0) P = 0.36

Baseline characteristics in PCOS OUH and risk of development of CVD

Hazard ratios are presented for crude models and models corrected for age and BMI

*BP* blood pressure, *LDL* low density lipoprotein, *HDL* high density lipoprotein, *2* *h*: 2 hours (during oral glucose tolerance test), *HOMA-ir* homeostasis model assessment of insulin resistance, SHBG: Sex hormone-binding globulin

^a^Except age which is adjusted for BMI alone, and BMI which is adjusted for age alone

## Discussion

In the present study, we demonstrate a higher incidence rate of CVD including dyslipidemia and hypertension in Danish women with PCOS compared to age-matched controls. The OR for development of CVD including hypertension and dyslipidemia was 1.7 in PCOS, and a baseline diagnosis of obesity, diabetes, infertility, Charlson index ≥ 1, and use of OCP were significant, independent predictors of CVD. In a representative subgroup of women with PCOS from our outpatient clinic, the risk of CVD was adversely affected by higher BMI, waist, BP, lipids, insulin, and glucose levels upon PCOS diagnosis, whereas baseline testosterone levels did not predict risk of CVD. The PCOS OUH cohort was relatively lean (median BMI 26.9 kg/m^2^) upon PCOS diagnosis and the average age was 29 years, but 22% women developed CVD during a median follow up of 11.1 years. When the diagnoses hypertension and dyslipidemia were excluded from the study outcome, the OR for development of CVD was 1.4 in PCOS and 7% women with PCOS developed CVD during follow up compared to 5% controls.

To our knowledge this is the first nationwide study to describe risk of developing CVD in a predominantly premenopausal study population of PCOS. We could confirm results from recent studies where the OR for CVD was increased in women with PCOS [5–8, 23, 24]. However, the present study design also allowed us to test the modifying effect of several risk factors for the development of CVD in PCOS.

### BMI and metabolic risk in PCOS

We found that the cardiovascular risk in PCOS was closely associated with BMI. In PCOS Denmark, a baseline ICD10 diagnosis of obesity resulted in a HR between 2.4 and 2.7 for CVD. Further, we noted that 24% of women in PCOS OUH that developed CVD were lean by the time of PCOS diagnosis. These results were in accordance with previous studies who reported that the risk of CVD and/or stroke was attenuated but not normalized in women with PCOS after adjusting for BMI [5, 7, 24]. Our results regarding BMI and CVD were somewhat different from data regarding the risk of T2D in lean women with PCOS, where the risk of T2D was < 1% at time of PCOS diagnosis [20, 25] and the risk of T2D development during follow up was not increased compared to age-matched controls [4, 10]. Therefore, our data suggest that prospective screening for CVD is indicated also in lean women with PCOS, whereas prospective OGTT, fasting glucose or HbA1c measurements may not be necessary.

### Blood pressure

Screening for metabolic syndrome includes measurement of BP, lipid status and assessment of glucose metabolism. In regression analyses, high baseline BP was an important predictor of CVD development. Furthermore, more than 30% women in PCOS OUH had BP ≥ 130/85 mmHg, and we previously reported a three times increased risk of the ICD10 code hypertension in PCOS Denmark vs. controls at baseline [18]. In a Chinese population of women with PCOS, women with hypertension had higher lipid, glucose, insulin, and HOMA-ir levels than women without hypertension also after adjusting for BMI [26], which suggested that elevated BP was a marker of metabolic risk. Our results support that BP should be measured in all women with PCOS irrespective of BMI.

### Lipids

Elevated TG levels ≥ 1.7 mmol/L were found in 18% women in PCOS OUH, and in multiple regression analyses TG, LDL and total cholesterol levels were the best predictors of development of CVD and remained significant after adjusting for age and BMI. However, 66% of women in PCOS OUH that developed CVD had baseline TG < 1.7 mmol/L, which supported that other risk markers than lipid profile determined CVD risk in PCOS. Previous studies estimated that 70% women with PCOS had borderline or high lipid levels [27], but dyslipidemia depends on for example ethnicity [28] and age [29]. The inclusion of a relatively lean and young study population of predominantly Nordic origin could have affected our study results. Our data support the importance of performing a baseline lipid profile in PCOS as risk assessment, but treatment with statins is rarely indicated in women < 35 years [12].

### Insulin resistance and glycemic status

HbA1c, fasting insulin, and HOMA-ir were higher at baseline evaluation in women in PCOS OUH with development of CVD compared to those not diagnosed with CVD. In multiple regression analyses, 2 h BG, fasting insulin, and HOMA-ir predicted development of CVD after adjusting for age and BMI, whereas HbA1c did not predict CVD risk. We could not confirm that HbA1c is a better predictor of CVD than fasting or 2 h glucose [30]. Median HbA1c was however relatively low in our study cohort, which could have affected study results. In PCOS Denmark, a baseline diagnosis of diabetes increased the risk of CVD more than threefold, confirming that women with PCOS and diabetes need special attention regarding risk of CVD.

### Age

Age was a significant predictor for CVD in the present study, but the effect was moderate as the median age at CVD diagnosis was only 1 year lower in PCOS Denmark vs. controls and 90% vs. 88% was aged < 50 years at CVD diagnosis. BMI and age are closely associated [29, 31], but the significant association between age and CVD development remained significant after adjusting for BMI. Our data could reflect that hypertension may be diagnosed at a relatively young age, especially as BP is measured in all women during pregnancy, but the exclusion of hypertension as a CVD outcome still resulted in a relatively low medium age at CVD diagnosis. Mani et al. [32] found a prevalence of 27% for myocardial infarction and angina in women with PCOS aged > 65 years attending a specialized clinic in United Kingdom and the OR for myocardial infarction was 12.9 in women with PCOS aged > 65 years vs. controls [32]. The risk for stroke was not significantly increased in PCOS in the meta-analysis by Anderson et al., though the risk for stroke became significantly increased in PCOS when only studies in women of average age > 45 years were included [6]. Increasing age in PCOS was associated with higher blood pressure, more adverse lipid profile, and impaired beta cell function [29]. Therefore, the diagnosis pattern could differ significantly in peri- and postmenopausal study populations of women with PCOS. We could test this hypothesis by repeating the present study after longer follow up duration.

### Androgen status

Testosterone levels at baseline did not predict development of CVD. Accordingly, we recently reported that the presence of individual Rotterdam criteria was not associated with cardio-metabolic diagnoses upon diagnosis in PCOS OUH despite a more adverse metabolic risk profile in women with polycystic ovaries and irregular menses [18]. Furthermore, testosterone levels did not predict the risk of T2D in PCOS OUH [10]. Importantly, mean BMI varies between different PCOS phenotypes [33, 34], and studies regarding vascular risk should adjust for BMI. Different results could be found in more obese study populations predominantly consisting of other PCOS phenotypes. Further prospective studies are needed to determine if the long-term cardiovascular risk is affected by PCOS phenotype independent of BMI.

### OCP

OCP is often used to treat hyperandrogenism and irregular menses in PCOS. We found that treatment with OCP increased the risk of CVD in PCOS Denmark, but this became non-significant when dyslipidemia and hypertension was not considered a CVD outcome. The most straightforward explanation would be that OCP is generally not prescribed for women with hypertension, but the register based design of the present study limits the possibility of firm conclusions regarding this point. The present study design did not allow us to adjust for use of OCP after the diagnosis of PCOS. However, our data confirmed increased risk of VTE in PCOS [16] and that the risk of CVD increased further during use of OCP [7, 17]. Metabolic side-effects of OCP are a matter of discussion. We and others reported increased insulin levels during OGTT in women with PCOS treated with OCP [35, 36] along with weight gain [14], which could increase the risk of development of CVD. Furthermore, treatment with OCP was associated with increased activation of the coagulation system [15], which was associated with increased thromboembolic risk [17]. The present data support that the possible benefit of OCP on PCOS-related symptoms needs to be balanced against possible metabolic side effects in each patient.

### Strengths and limitations

An important strength of this study was the nationwide data and the embedded cohort of PCOS OUH with available clinical and biochemical data, which allowed us to test hypotheses that could not be evaluated in the national cohort. Limitations to the study include the following. Women were diagnosed with PCOS through hospital contacts, hence some women in the control group may have undiagnosed PCOS, which could lead to underestimation of relative CVD risk in PCOS. However, undiagnosed PCOS likely represents milder cases. The Rotterdam criteria were introduced in 2003 [37], which implied the inclusion of more mild phenotypes as part of the PCOS definition. The use of different definitions of PCOS is a limitation of the present study. Furthermore, the study was conducted in a relatively lean and predominantly young Nordic study population with low baseline risk of CVD. Some ascertainment bias is possible, especially for potentially asymptomatic circulatory conditions such as hypertension and dyslipidemia, with PCOS patients potentially having more BP and lipid measurements done than population controls. The present study results need to be reproduced in study populations consisting of other phenotypes and with higher baseline metabolic risk. Our definition of CVD as an outcome was relatively broad and included both prevalent conditions such as hypertension and incident events such as myocardial infarction. We addressed this by defining a secondary study outcome without hypertension and dyslipidemia, and through tabulating the individual disease outcomes.

The present study design included baseline clinical and biochemical characteristics as predictors of CVD risk. PCOS is associated with higher serum levels of IL-6 and other inflammatory cytokines [2, 38], which could be associated with increased risk of CVD. Metabolic risk in PCOS could be related to IL-6 gene polymorphism [39] and the metabolic risk may be modified by lifestyle intervention [40] and metformin treatment [41]. More studies are needed regarding the long term effect of lifestyle and medical intervention on CVD risk in PCOS. Potential improvements to cohort studies like this could include collection of more sophisticated measures of baseline inflammatory markers and prospective measures of for example BMI and glycemic status.

## Conclusion

The risk of development of CVD was significantly increased in PCOS with hypertension as the most common cardiovascular diagnosis. The cardiovascular risk was predicted by baseline age and screening for the elements of the metabolic syndrome (BMI, BP, lipid status, and glucose), and our data support that the risk of developing CVD must be considered even in young, lean women with PCOS. The risk of CVD was adversely affected by the use of OCP.

## Additional file

**Additional file 1.** Assays in PCOS OUH: Details regarding applied assays in the PCOS OUH cohort.

## Abbreviations

BG: blood glucose
BP: blood pressure
CI: confidence interval
CVD: cardiovascular disease
HOMA-ir: homeostasis model of insulin resistance
HR: hazard ratio
OCP: oral contraceptive pills
OGTT: oral glucose tolerance test
NPR: national patient register
PCOS: polycystic ovary syndrome
PY: person years
TG: triglycerides
VTE: venous thromboembolism
