# Supplementary material for: Cardiovascular disease in a nationwide population of Danish women with polycystic ovary syndrome
Source: Cardiovasc Diabetol. 2018 Mar 8;17:37. doi: 10.1186/s12933-018-0680-5 (PMC5844097; doi:10.1186/s12933-018-0680-5)
Supplement: Supplementary file 1 — Additional file 1. Assays in PCOS OUH: Details regarding applied assays in the PCOS OUH cohort. [file 12933_2018_680_MOESM1_ESM.docx]

**Assays in PCOS OUH**

Serum total testosterone was analyzed using a specific radioimmunoassay after extraction as previously described ^1^ and sex hormone-binding globulin (SHBG) was analyzed by an AutoDelfia assay. The intra-assay coefficient of variation (CV) for the total testosterone assay was 8.2% and 5.2% for SHBG. The inter-assay CV for the total testosterone assay was 13.8% and 7.5% for SHBG. Insulin was analyzed by a time-resolved fluoroimmunoassay using a commercial kit (AutoDelfia, Wallac Oy, Turku, Finland) with an intra-assay variation 2.1-3.7% and inter-assay variation 3.4-4.0%. Plasma total cholesterol, high-density lipoprotein (HDL) cholesterol, and triglycerides (TG) were analyzed by enzymatic colorimetric reactions (Modular P, Roche), while low-density lipoprotein (LDL) cholesterol was calculated using the Friedewald equation. Blood glucose was measured on capillary ear blood using Hemo Cue. We calculated the homeostasis model assessment of insulin resistance (HOMA-ir = fasting insulin x fasting blood glucose / 22.5).^2^ HbA1c was measured by high-performance liquid chromatography as fraction of total haemoglobin A0 using Tosoh G8 (Medinor, Broendby, Denmark) with reagents as recommended by the supplier. The analytical CV was 0.9%. During 1997-2003, an oral glucose tolerance test (OGTT) was part of the routine evaluation program for newly referred women with PCOS. ^3^ Capillary blood glucose was measured at fasting and 30, 60, and 120 minutes after oral ingestion of a glucose load containing the equivalent of 75 g anhydrous glucose dissolved in water.

**Reference List**

1 Lykkesfeldt G, Bennett P, Lykkesfeldt AE, Micic S, Moller S, Svenstrup B. Abnormal androgen and oestrogen metabolism in men with steroid sulphatase deficiency and recessive X-linked ichthyosis. Clin Endocrinol (Oxf) 1985; 4: 385-93.

2 Radziuk J. Insulin sensitivity and its measurement: structural commonalities among the methods. J Clin Endocrinol Metab 2000; 12: 4426-33.

3 Glintborg D, Henriksen JE, Andersen M, et al. Prevalence of endocrine diseases and abnormal glucose tolerance tests in 340 Caucasian premenopausal women with hirsutism as the referral diagnosis. Fertil Steril 2004; 6: 1570-9.

**Appendix Table S1. Baseline characteristics in women with PCOS and controls**

|  | |  | | **PCOS OUH** | **PCOS Denmark** | **Controls** |  |  |
| --- | --- | --- | --- | --- | --- | --- | --- | --- |
|  |  |  |  | N=1,159 | N=17,995 | N=52,329 | P^a^ | p^b^ |
| Age (y) at PCOS diagnosis, median (Q1 – Q3) | | | | 29 (22-35) | 29 (23-35) | 29 (23-35) | 0.31 | 0.02 |
| min-max |  | | | 12-54 | 12-60 | 12-60 |  |  |
| **ICD10 codes present before index date** | | | | N (%) | N (%) | N (%) |  |  |
| Obesity | | | E66 | 148 (13) | 2,005 (11) | 648 (1) | 0.07 | <0.001 |
| Type 1 diabetes | | | E10, E13 | 5 (0.4) | 97 (0.5) | 194 (0.4) | 0.61 | 0.002 |
| Type 2 diabetes | | | E11, E14 | 16 (1) | 198 (1) | 104 (0.2) | 0.34 | <0.001 |
| Gestational diabetes | | | O24 | 13 (1) | 243 (1) | 152 (0.3) | 0.58 | <0.001 |
| Diabetes total | | | E10,11,13,14, O24 | 30 (3) | 448 (3) | 367 (1) | 0.82 | <0.001 |
| Infertility | | | N97, Z350 | 192 (17) | 4,158 (23) | 2,007 (4) | <0.001 | <0.001 |
| Number of births | | | 0 | 895 (77) | 14,500 (81) | 45,564 (87) | 0.01 | <0.001 |
|  | | | 1 | 168 (15) | 2,279 (13) | 3,985 (8) |  |  |
|  | | | 2 | 83 (7) | 1,002 (6) | 2,280 (4) |  |  |
|  | | | ≥3 | 13 (1) | 214 (1) | 500 (1) |  |  |
| Comorbidity | | | | 42 (4) | 796 (4) | 1,646 (3) | 0.17 | <0.001 |
| **Medicine prescriptions filled before index date (ever use)** | | | | |  |  |  |  |
| Antidiabetics | A10 | | | 83 (7) | 2,103 (12) | 355 (0.7) | <0.001 | <0.001 |
| Oral contraceptives | G03AA, G03AB G03HB01 | | | 654 (56) | 10,432 (58) | 16,005 (31) | 0.27 | <0.001 |
| Fertility treatment | G03GA, G03GB, N04BC | | | 154 (13) | 3,107 (17) | 1,060 (2) | <0.001 | <0.001 |

Comorbidity was defined as a Charlson index ≥1.

p^a^ Between PCOS OUH and the remainder of PCOS Denmark.

p^b^ Between PCOS Denmark and controls.

Chi-square test (for categorical variables) and non-parametric test on the equality of medians (for continuous variables).

**Appendix Table S2. Baseline clinical and biochemical data in the PCOS OUH patient cohort (N=1,159)**

|  | **N (%)** | **Median (Q1 – Q3)** |
| --- | --- | --- |
| Age (years) | 1,159 (100) | 29 (22 – 35) |
| BMI (kg/m^2^) | 1,076 (93) | 27.0 (23.0 – 32.4) |
| Waist (cm) | 721 (62) | 88 (78 – 103) |
| Systolic BP (mmHg) | 773 (67) | 124 (115 – 134) |
| Diastolic BP(mmHg) | 772 (67) | 80 (71 – 87) |
| LDL cholesterol (mmol/L) | 847 (73) | 2.7 (2.2 – 3.3) |
| HDL cholesterol (mmol/L) | 849 (73) | 1.4 (1.1 – 1.6) |
| Cholesterol (mmol/L) | 860 (74) | 4.6 (4.1 – 5.3) |
| Triglycerides (mmol/L) | 848 (73) | 1.0 (0.7 – 1.5) |
| Prolactin (µg/L) | 960 (83) | 7 (5 – 10) |
| HbA1c (mmol/mol) | 571 (49) | 34 (31 – 37) |
| Fasting blood glucose (mmol/L) | 534 (46) | 4.6 (4.3 – 5.0) |
| 2h blood glucose (mmol/L) | 522 (45) | 6.1 (5.2 – 7.1) |
| Fasting insulin (pmol/L) | 567 (49) | 55 (38 – 90) |
| HOMA-ir (pmol mmol L^-2^) | 540 (47) | 12.2 (8.0 – 20.1) |
| Total testosterone (nmol/L) | 825 (71) | 1.78 (1.26 – 2.40) |
| SHBG (nmol/L) | 1,073 (93) | 44 (31 – 66) |
| Free testosterone (nmol/L) | 811 (70) | 0.033 (0.021 – 0.050) |
| BMI ≥25 kg/m^2^ | 655 (61) |  |
| Waist ≥88 cm | 371 (52) |  |
| Systolic BP ≥130 mmHg | 289 (38) |  |
| Diastolic BP ≥85 mmHg | 263 (34) |  |
| Triglycerides ≥1.7 mmol/L | 156 (18) |  |

BP: Blood pressure, LDL: low density lipoprotein, HDL: High density lipoprotein, 2h: 2 hours (during oral glucose tolerance test), HOMA-ir: Homeostasis model assessment of insulin resistance, SHBG: Sex hormone-binding globulin.
